# Access to drinking safe water and its associated factors among households in East Africa: a mixed effect analysis using 12 East African countries recent national health survey

**DOI:** 10.1186/s41043-024-00562-y

**Published:** 2024-05-24

**Authors:** Bewuketu Terefe, Mahlet Moges Jembere, Nega Tezera Assimamaw

**Affiliations:** 1 Department of Community Health Nursing, School of Nursing, College of Medicine and Health Sciences, University of Gondar, Gondar, Ethiopia; 2 Department of Emergency and Critical Care Nursing, School of Nursing, College of Medicine and Health Sciences, University of Gondar, Gondar, Ethiopia; 3 Department of Pediatrics and Child Health, School of Nursing, College of Medicine and Health Sciences, University of Gondar, Gondar, Ethiopia

**Keywords:** Access to drinking water, Factor, Households, East Africa, Mixed effect analysis

## Abstract

**Background:**

More than half of the population in Sub-Saharan Africa (SSA) faces limited access to safe drinking water. Unimproved water sources can pose risks to the health of entire households, particularly women and children. Despite the fact that East African countries have some of the poorest drinking water infrastructures globally, there is a lack of published data on this issue. Consequently, the objective of this study was to examine access to safe drinking water and its determinants among households in East Africa, utilizing recent nationally representative data.

**Methods:**

This study analyzed data from recent demographic and health surveys conducted in 12 East African nations between 2011 and 2022. Data were gathered from 204,275 households. A stratified two-stage cluster sampling method was employed, with enumeration areas serving as the main sampling units and households serving as the secondary sampling units. Binary and multiple multilevel logistic regression were used to examine the relevant factors associated with the use of different sources of drinking water in the region. In binary regression and multiple regression, P values of ≤ 0.2 and < 0.05, respectively, were used to determine the statistical significance of variables in the final model.

**Results:**

Approximately 72.62% (95% CI = 72.43, 72.83) of households have utilized improved sources of drinking water. Household heads aged 25–35 years (AOR = 1.09, 95% CI = 1.04, 1.14), 36–45 years (AOR = 1.09, 95% CI = 1.04, 1.14), and > 45 years (AOR = 1.08, 95% CI = 1.04, 1.14), those with secondary/higher education (AOR = 1.24, 95% CI = 1.20–1.29), and individuals in wealth index categories of poorest (AOR = 0.17, 95% CI = 0.16, 0.18), poorer (AOR = 0.21, 95% CI = 0.19, 0.22), middle (AOR = 0.25, 95% CI = 0.24, 0.27), and richer (AOR = 0.36, 95% CI = 0.34, 0.38) were associated with improved sources of drinking water. Additionally, female household leaders (AOR = 1.23, 95% CI = 1.20, 1.26), > 30 min of time taken to access the water source (AOR = 2.00, 95% CI = 1.95, 2.05), improved toilet facilities (AOR = 2.25, 95% CI = 2.19, 2.31), rural residence (AOR = 0.43, 95% CI = 0.42, 0.45), high community wealth (AOR = 1.31, 95% CI = 1.13–1.51), community media exposure (AOR = 1.32, 95% CI = 1.15, 1.51) were associated with improved sources of drinking water, respectively.

**Conclusion:**

Approximately three-quarters of the population in East Africa has access to improved drinking water, although the quality of water in the region is still considered poor. It is important for relevant organizations to collaborate in order to improve the quality of drinking water, with special attention given to high-risk groups such as communities with high poverty and low literacy rates, poor households, and rural residents. Strengthening women’s empowerment and increasing mass media exposure can also play a crucial role in accelerating the adoption of improved drinking water sources in East Africa.

## Introduction

A clean environment, excellent health, and a healthy nation require high-quality water [1–3]. However, inadequate access to clean drinking water, unimproved sanitation facilities, poor hygiene habits, and ineffective water management practices can all contribute to the development of waterborne diseases, which account for 6.3% of all global fatalities [4, 5]. According to evidence, unimproved drinking water and sanitation are responsible for around 50% of all diarrheal deaths globally [5].

From the launch of the Millennium Development Goals (MDGs) in 1990 to their expiration in 2015, there was an increase in the use of improved water sources globally (from 76 to 91%). Nonetheless, despite coordinated efforts, much remains to be done in terms of water access [6]. After the MDGs were phased out in September 2015, the world turned its attention to a new plan known as the Sustainable Development Goals (SDGs) [7, 8]. The SDGs’ Target 6 is to ensure universal and equitable access to clean and cheap drinking water for all during the next 15 years [7]. In 2015, the United Nations set the Sustainable Development Goal of providing equitable access to safe and affordable drinking water for all by 2030 [9]. Lower morbidity has been linked to greater access to water and sanitation. Water, Sanitation, and Hygiene (WASH) are major public health concerns in Sub-Saharan Africa (SSA) with relatively poor coverage [10]. Low-income households are the most vulnerable during times of scarcity [11]. Water and sanitation services continue to differ greatly between urban and rural regions, particularly in SSA [12].

It is estimated that 80% of people in low- and middle-income countries lack access to basic WASH services [5]. Children in Sub-Saharan Africa must travel to wells instead of school due to a lack of access to clean water [13, 14]. This demonstrates the magnitude of the problem. As a result, access to an adequate supply of drinkable water is critical for promoting public social welfare and development.

The universal access to safe drinking water is critical for promoting population health and well-being [15]. Using unsafe water sources raises the risk of cholera, typhoid, dysentery, schistosomiasis, salmonellosis, and infections of the respiratory, skin, and eye systems [5, 16, 17]. Infections such as helminthiasis, scabies, and trachoma can also emerge when there is a lack of access to clean water [5]. According to the 2017 WHO progress report, and other evidences on sanitation and drinking water, over half of East Africans rely on unimproved water sources [13, 18, 19]. Despite the fact that enhanced water access is a significant public health concern, to the best of our knowledge, there is currently no documented data on it in the region. Therefore, this study utilized recent nationally representative data to investigate the source of drinking water and its associated characteristics in East Africa. The findings of this study will aid stakeholders, researchers, and policymakers in implementing strategies to mitigate the consequences of using water from unimproved sources in the region.

## Methods

### Study setting, and period

The most recent Demographic and Health Survey (DHS) dataset for East African nations during a ten-year period (2011–2022) provided the information. A standardized dataset was employed [20] “To collect a substantial and representative sample size from the population source and consider all relevant factors, Demographic and Health Surveys (DHS) are conducted. These surveys gather comparable data on a global scale, employing large sample sizes that are population-based and nationally representative for each nation [20]. The 14 nations that make up Eastern Africa are spread throughout the Horn of Africa, the Indian Ocean islands, and the Great Lakes region. These nations struggle with comparable economic, social, and environmental problems, and they worry that they won’t achieve all of the Sustainable Development Goals’ (SDGs) objectives [21]. “East Africa is the region of the African continent that spans the Horn and the eastern parts of the Sahara Desert. It is estimated to be home to 486,766,759 people and covers an area of 6,667,493 square kilometers (2,574,332 square miles), constituting 6.03% of the world’s population.

### Data source and study population

We used DHS data from the last ten years, spanning from 2011 to 2022. Although DHS surveys were conducted in 14 Eastern countries between 2011 and 2022, only around 12 of them were considered for our study since the remaining surveys lacked information on the outcome variable. After incorporating the data from each country, a total weighted sample of 204,275 households interviewed for the source of drinking water access was used for the final analysis (Table 1).

**Table 1:** Countries, sample size, and survey year of Demographic and Health Surveys included in the analysis for 12 East African countries

Country	Survey year	Sample size(weighted)	Frequency(weighted)
Burundi	2016/17	15,977	7.82
Ethiopia	2016	16,650	8.15
Comoros	2012	4,4821	2.19
Kenya	2022	37,911	18.56
Madagascar	2021	20,510	0.04
Malawi	2016/17	26,361	12.90
Mozambique	2011	13,919	6.81
Rwanda	2015/16	12,949	6.34
Tanzania	2015	12,563	6.15
Uganda	2016	19,588	9.59
Zambia	2018	12,831	6.28
Zimbabwe	2015	10,534	5.16

### Sample size determination and sampling method

Demographic and Health Survey reports were available for approximately 12 out of the 13 nations in East Africa. The most current conventional census frame was used in each of the surveys conducted in the nations listed below. Demographic and health survey samples are typically divided into urban and rural areas within each administrative geographic region. In the first round of sampling, Enumeration Areas (EAs) were chosen with a probability proportional to their size within each stratum. The systematic sampling approach was then used to select a defined number of households from the designated EAs in the second step of sampling. Following the household listing, equal probability systematic sampling was employed to select a certain number of households from within the defined cluster [20].

### Variables of the study

#### The outcome variable

The outcome variable in this study was access to safe/improved drinking water sources among households and the de jure population in East Africa. The sources of drinking water were categorized into two groups: improved and unimproved sources. The improved sources of drinking water included piped water into the dwelling, piped water to the yard/plot, public tap/standpipe, piped water to the neighbor, tube well or borehole, protected well, protected spring, rainwater, tanker truck, cart with a small tank, and bottled water. On the other hand, unimproved drinking water sources included unprotected wells, unprotected springs, surface water (such as rivers, dams, lakes, ponds, streams, canals, and irrigation channels), and other sources. The outcome variable was then recategorized as “Yes” (coded as “1”) if the household used an improved source of drinking water, and “No” (coded as “0”) if the household consumed an unimproved source of drinking water. This classification and analysis were done according to the guide to DHS statistics book [22].

#### The independent variables

Independent variables included household-level and sociodemographic factors. These factors encompassed the age and sex of the household head, educational status, types of places of residence, marital status, household wealth index, current employment status, exposure to mass media, household size, number of children under five, number of adults, time taken to access water sources, types of toilet facilities, community-level literacy, country, and community-level wealth.

### Operational definitions

#### Improved/unimproved toilet facility types

This variable was identified as follows for improved toilet facility types: flush - to piped sewer system, flush - to septic tank, flush - to pit latrine, flush - don’t know where, pit latrine - ventilated improved pit (VIP), pit latrine - with slab, and composting toilet. However, for unimproved toilet facility types, the categories used were flush - to somewhere else, pit latrine - without slab / open pit, bucket toilet, hanging toilet/latrine, and others.

#### Community household head’s education

The educational achievement of household heads in a community is reflected in the median distribution of educational attainment. If the proportion of household heads in the community with at least a secondary education is below the median (57%), it is considered low. Conversely, if it exceeds the median (58–100%), it is considered high.

#### Community household head media exposure

The media exposure variable was based on individual responses to media exposure through radio, books/newspapers, or television. A low level of media exposure was defined as the proportion of household heads in the community who reported media exposure between 0% and 49%. On the other hand, a high level of media exposure was defined as the proportion between 50% and 100%.

#### Community household head wealth

The same process was used to determine this variable based on the wealth index of each household. In the two lowest quintiles of wealth in a community, the variable was considered high if between 64% and 100% of women were present, and low if between 0% and 63% of women were present in the household.

### Data management and statistical analysis

The variables in the study were extracted, cleaned, and recoded using STATA version 17. During any statistical analysis, the data were weighted using sample weights to account for the unequal probability of selection due to the sampling process used in DHS data. This ensured the representativeness of the survey results. To account for the hierarchical nature of the data, a two-level multilevel fixed effect binary and multiple logistic regression analysis was used to assess the effect of explanatory variables on access to drinking water among East African households. The data is divided into two levels: a group of J EAs and within-group j (j = 1, 2…J), and a random sample nj of level-one units (households). The response variable is represented by as; Y_ij_ = 0 if the i^th^ households was in the j^th^ EA’s had source of drinking water 1 if i^th^ households was in the j^th^ EAs had no exposure of access to drinking improved water.

To account for the nested effect, acceptable deductions and conclusions from this data require adequate modeling techniques such as multilevel modeling, which includes variables assessed at multiple levels of the hierarchy [23]. Four models were fitted to the data. The initial model used to calculate the extent of cluster variation in abortion was an empty model with no explanatory parameters. To compute differences between clusters (EAs), the intra-class correlation coefficient (ICC), proportional change in variance (PCV), and median odds ratio (MOR) were employed. The ICC represents the fraction of variance explained by the population grouping structure. Unlike the null model, the PCV assesses the total variation attributable to individual and community-level components in the multilevel model [24].

The MOR is defined as the median value of the odds ratio between the clusters at high and low risk of salt iodization when two clusters (EAs) are chosen at random. The second model only included community-level variables, the third model only included individual-level variables, and the fourth model included both individual and community-level variables. The model with the lowest deviation (-2LLR) was chosen as the best-fitted model for the data. In the bivariable analysis, variables with a p-value of 0.2 were considered for the multivariable analysis. The multivariable multilevel binary logistic model presented the Adjusted Odds Ratio (AOR) with a 95% confidence interval to determine the factors related to the source of drinking water among households. The statistical significance of the final model was set at *p* < 0.05.

## Results

### Sociodemographic characteristics of study participants

This survey comprised weighted samples from 204,275 households. In terms of age, approximately 81,369 (39.83%) of the household heads were over 45 years old. Regarding place of residence and marital status, the majority, 149,541 (73.21%), and 144,546 (70.76%) lived in rural areas and were married, respectively. In terms of educational level, 93,999 (46.05%) of the participants had completed primary school. Media exposure was reported by 109,617 (53.65%) of the survey participants. Approximately 141,777 (69.40%) of households were headed by males.

In terms of time to access drinking water sources and toilet facility types, slightly over half, 102,476 (50.17%), took more than 30 min to access drinking water sources, and almost half, 106,882 (52.32%), had toilet facilities that required more than 30 min to access. The majority of households, 166,281 (81.37%), had fewer than six family members. Similarly, approximately 113,998 (55.81%) households had at least one adult and 108,122 (52.93%) households had under five children. Regarding community-level characteristics, about 139,307 (68.20%), 152,962 (74.80%), and 109,565 (53.64%) households showed low community-level education, mass media exposure, and wealth index, respectively. About 42,927 (21.01%) households belonged to the richest wealth quantile (Table 2).

**Table 2:** Sociodemographic and household related characteristics of source of drinking water access among households in East Africa (*n* = 204,275)

Variables on source of drinking water access	Frequency	Percentage
**Household head age**		
< 25	16,498	8.08
25–35	59,112	28.94
36–45	47,297	23.15
> 45	81,369	39.83
**Marital status**		
Never married	13,120	6.42
Married	144,546	70.76
Divorced/widowed	46,610	22.82
**Educational status**		
No formal education	47,719	23.38
Primary	93,999	46.05
Secondary and higher	62,402	30.57
**Household wealth index**		
Poorest	39,994	19.58
Poorer	39,637	19.40
Middle	39,539	19.36
Richer	42,178	20.65
Richest	42,927	21.01
**Mass media exposure**		
No	94,658	46.34
Yes	109,617	53.65
**Sex of the household head**		
Male	141,777	69.40
Female	62,498	30.60
**Toilet facility type**		
Unimproved	97,393	47.68
Improved	106,882	52.32
**Time to get water source in minutes**		
≤ 30	101,799	49.83
> 30	102,476	50.17
**Household members**		
1–6	166,2281	81.37
> 6	38,047	18.63
**Number of under five children**		
No	96,153	47.07
Yes	108,122	52.93
**Number of adults**		
No	90,277	44.19
Yes	113,998	55.81
**Types of residence**		
Urban	54,734	26.79
Rural	149,541	73.21
**Community-level wealth**		
Low	109,565	53.64
High	94,710	46.36
**Community-level mass media exposure**		
Low	152,962	74.88
High	51,313	25.12
**Community level literacy**		
Low	139,307	68.20
High	64,968	31.80

### Random effect analysis

The random-effects model revealed significant clustering of the source of improved drinking water across communities (community level variance (CLV) = 1.91). The ICC score of the null model indicated that cluster variability accounted for 36.65% of the overall variation in drinking water source utilization. The null model’s MOR value of 3.72 suggests that there is variation in the use of improved drinking water sources among clusters. In other words, households in the cluster with the highest proportion of safe drinking water consumption had a 3.72 times greater risk of using an improved source of drinking water compared to their counterparts. As shown in Table 3, the PCV increases from 47.12% (Model I) to 45.03% (Model III), indicating that Model III best captures the diversity of improved drinking water sources. Model III also has the lowest Deviance and AIC, making it the model with the best fit (Table 3).

**Table 3:** Individual and community-level factors associated with source of drinking water access among households in East Africa (*n* = 204,275)

Variables on source of drinking water access	Null model	Model I	Model II	Model III
		AOR (95% CI)	AOR (% CI)	AOR (95% CI)
**Household head age**				
< 20		1		1
20–35		1.08(1.03,1.12)		1.09(1.04,1.14) *
36–45		1.07(1.02,1.12)		1.09(1.04,1.14) *
> 45		1.06(1.01,1.11)		1.08(1.04,1.13) *
**Educational status**				
No formal education		1		1
Primary		1.02(0.99,1.04)		1.01(0.98,1.04)
Secondary and higher		1.31(1.26,1.36)		1.24(1.20,1.29) *
**Household wealth index**				
Poorest		0.11(0.11,0.12)		0.17(0.16,0.18) *
Poorer		0.14(0.13,0.15)		0.21(0.19,0.22) *
Middle		0.17(0.16,0.19)		0.25(0.24,0.27) *
Richer		0.28(0.26,0.30)		0.36(0.34,0.38) *
Richest		1		1
**Mass media exposure**				
No		1		1
Yes		0.99(0.95,1.04)		0.95(0.91,1.00)
**Sex of the household head**				
Male		1		1
Female		1.26(1.23,1.30)		1.23(1.20,1.26) *
**Time to get water source**				
≤ 30 min		1		1
> 30 min		2.13(2.07,2.19)		2.00(1.95,2.06) *
**Toilet facility type**				
Unimproved		1		1
Improved		2.28(2.22,2.34)		2.25(2.19,2.31) *
**Household member**				
1–6		1		1
> 6		0.93(0.91,0.96)		0.95(0.92,0.97)
**Types of residence**				
Urban			1	1
Rural			0.14(0.14,0.15)	0.43(0.42,0.45) *
**Community-level wealth**				
Low			1	1
High			1.35(1.183,1.55)	1.14(0.99,1.29)
**Community level literacy**				
Low			1	1
High			1.49(1.28,1.73)	1.31(1.13,1.51) *
**Community mass media**				
Low			1	1
High			1.51(1.31,1.74)	1.32(1.15,1.51) *
**Random parameters and model comparison**				
Community-level variance	1.91	1.01	1.22	1.05
ICC (%)	36.65	25.15	27.05	24.44
MOR (%)	3.72	2.72	2.86	2.65
PCV	Reference	47.12	36.13	45.03
Log-likelihood (LLR)	-112541.77	-95917.88	-104205.85	-94979.61
DIC (-2LLR)	225,083.54	191,835.76	208,411.7	189,959.22
AIC	225087.5	191867.8	208423.7	189999.2

*Indicates significance at p-value < 0.05 variables in final model regression

### Factors associated with source of safe drinking water among households in East Africa

Bivariable and multivariable multilevel logistic regression analyses were used to identify factors associated with the source of drinking water among households. In the binary model analysis, variables such as age and gender of the household head, educational level, toilet types, time to access the water source, wealth index, media exposure, source of drinking water, toilet facility types, family size, type of residence, community mass media exposure, community wealth, and community level were associated or selected as candidate variables for the final model with iodized salt use in East Africa (*P* ≤ 0.2). The final model indicated that the age and gender of the household head, educational level of the household head, wealth index, time to access the drinking water source, toilet facility types, type of residence, community wealth, and community-level mass media exposure were significantly associated with an improved source of drinking water (*p* < 0.05).

Household heads aged 25–35 years, 36–45 years, and over 45 years had 1.20, 1.16, and 1.18 times higher chances of using an improved water source for drinking compared to youth household heads (AOR = 1.09, 95% CI = 1.04, 1.14), 36–45 years (AOR = 1.09, 95% CI = 1.04, 1.14), and over 45 years (AOR = 1.08, 95% CI = 1.04, 1.14). Household heads with secondary/higher education had 1.24 times higher chances of accessing an improved source of drinking water compared to those with no formal education (AOR = 1.24, 95% CI = 1.20–1.29).

Households with the poorest, poorer, middle, and richer wealth indexes were 83%, 79%, 75%, and 64% less likely, respectively, to use a safe/improved source of drinking water than the richest households (AOR = 0.17, 95% CI = 0.16, 0.18), (AOR = 0.21, 95% CI = 0.19, 0.22), (AOR = 0.25, 95% CI = 0.24, 0.27), and (AOR = 0.36, 95% CI = 0.34, 0.38). Households led by female household heads had 1.23 times higher odds of using an improved source of drinking water compared to those led by male household heads (AOR = 1.23, 95% CI = 1.20, 1.26). Households with a time of more than 30 min to access a drinking water source and those using better toilet facility types had 2.00- and 2.25-times higher odds of using an improved source of drinking water, respectively, compared to those with less than 30 min and unimproved water and toilet facilities (AOR = 2.00, 95% CI = 1.95, 2.05) and (AOR = 2.25, 95% CI = 2.19, 2.31). Regarding community-level variables, rural residents (AOR = 0.43, 95% CI = 0.42, 0.45), households with high community wealth, and high media exposure had 1.31- and 1.32-times higher probabilities of using an improved source of drinking water, respectively (AOR = 1.31, 95% CI = 1.13–1.51) and (AOR = 1.32, 95% CI = 1.15, 1.51) (Table 3).

## Discussion

The goal of this study was to identify determinants of drinking water source in East Africa. Approximately three-quarters of East African households have access to improved drinking water sources. The findings were consistent with those of an Ethiopian investigation [25]. However, it is lower than in Eswatini [26], Zambia [27], and Ghana [28]. The gap could be attributed to socioeconomic factors, variable types, sample sizes, and model choices utilized in the studies, as well as variations between nations and discrepancies in the study periods. This comparison highlights potential disparities in water access across different nations, enabling policymakers to identify areas in need of improvement and implement strategies to enhance access to safe drinking water. It suggests that multiple factors influence water access and should be considered when designing public health interventions or policies pertaining to water supply. Socioeconomic inequalities and variations in water source types underscore the importance of addressing fairness and tailoring interventions to specific contexts. Additionally, the sentence indicates a need for further research to gain a better understanding of the factors that influence drinking water sources in East Africa. Conducting more rigorous studies with larger sample sizes and employing appropriate models can offer more precise insights into the factors that impact water access. This knowledge can then inform evidence-based interventions and policies aimed at enhancing access to safe drinking water in the region.

Household heads with ages of 25–35 years, 36–45 years, and more than 45 years had better chances of using improved water source for drinking as compared to youth household heads. The findings are congruent with those of a study conducted in Ethiopia [29], and Vietnam [30]. One probable explanation is that those household heads over the age of 45 are more likely to be employed and thus have a higher chance of affording the expense of safe drinking water. Older adults may also have better social attraction and communication networks, as well as lived experience with improved or unimproved water resources. Furthermore, elderly household heads may be more aware of their well-being and may be more likely to use amenities that promote their health [28, 30].

Household heads with secondary/higher education, and community education level had 1.24, and1.31 times the chances of accessing improved source of drinking water compared to those with no formal education and low community level education respectively. It corresponded to research conducted in low, and middle income countries [31], Ethiopia [29, 32], Indonesia [33], Nigeria [34], and Ghana [35]. The possible reason for this association is that, because education is such a powerful tool, it is expected that the level of awareness about the health benefits of using a safe drinking water source will rise as the community’s literacy level rises, and more and more educated communities will have better access to safe drinking water infrastructure [31, 36]. Another possibility is that households with low community literacy are less aware of how to protect their available water supply [32].

Households with poorest, poorer, middle, and richer wealth indexes were 83% and 79%,75%, and 64% less likely, respectively, to use safe/improved source of drinking water than richest households. The findings are comparable to those of previous studies conducted in Ethiopia [29], Ghana [35], and Indonesia [33]. The rich may be able to afford the cost of safe drinking water, whereas the poor are disproportionately disadvantaged in the distribution of public services [25]. The reason for this is that affluence improves the ability to pay for municipal services like water and sanitation even when the local authority or government does not provide these services.

Communities from high media exposure had also revealed greater probabilities of using improved source of drinking water as compared to their counterparts. Documents from Tanzania [37], and Ethiopia [38] have found similar association between mass media exposure, and source of drinking water. One possible explanation for this could be that continuous and informational media exposure may produce potential health risks as a result of enhanced cleanliness. Our data suggest that exposure to mass media can increase source of drinking water, handwashing knowledge and practices following a mass media campaign. This potential strategy can be used to enhance water, sanitation, and hygiene habits on a large scale and should be tested in other scenarios in each country, particularly for rural inhabitants.

Households led by female household leaders had more times of using improved source of drinking water as those led by male household heads. This finding was applicable to other similar research in Vietnam [39], and African countries [40, 41]. This study underlined the importance of women in guaranteeing the family’s access to improved water sources, and as a result, they should be involved in enhanced water promotion programs at all levels and regions, as indicated in Côte d’Ivoire [42]. In many SSA households, women are in charge of WASH, as well as cooking and other household responsibilities. This direct link to water and sanitation shows that women may pay greater attention to such concerns than males, especially when women are the household leaders [40].

Households with greater than 30 min time to get source of drinking water, and who used better toilet facility types had shown higher odds of using improved source of drinking water respectively than those with less than 30 min, and unimproved water and toilet facility. According to an Ethiopian study, households with better drinking water sources were 1.37 times more likely to have access to improved toilet facilities than their counterparts [25]. A possible explanation is that a lack of access to proper sanitation is linked to restricted access to water supply, and households with improved water sources may practice more hygiene and sanitation [43]. The relationship between time to get a source of drinking water and water improvement was positively associated, although it contradicted studies done in Ethiopia [25, 44]. These studies put their justification like this is not surprising given that one of the reasons for WASH service inaccessibility is physical distance. However, in our opinion, because there are/might be few purchasable pipelines or better water sources in East Africa, individuals may have to travel a long distance to acquire this water.

Rural residential households with high community wealth and high media exposure had shown1.31 times greater probabilities of using improved source of drinking water. The findings are comparable with those of studies conducted in Ethiopia [29, 32], and Nigeria [34]. People in rural areas generally have low socioeconomic position, lack education, have a negative attitude, and adhere to other cultural or religious conventions, making it difficult for them to access improved drinking water [25, 42]. Because the bulk of the African population lives in rural locations far from basic infrastructure, it is preferable to include them in the public health programs [36].

The study had several merits. Firstly, it utilized a weighted nationally representative large dataset, which enhanced the generalizability of the findings. Secondly, the study employed a sophisticated model that accounted for the hierarchical structure of the data. Additionally, this study provides the first insight into the region of East Africa. Although the study included essential individual and community-level factors, it did not conduct a spatial analysis to identify significant hotspot locations. This could be a potential area of focus for future researchers in the field. Furthermore, the study did not control for the influence of available services on water and sanitation facilities in the analysis. It should be noted that service availability varied between countries, with more accessible and affordable services found in delta areas. This limitation should be considered when interpreting the results. Moreover, as it was a secondary data study, certain predictive variables of drinking water sources were not available in the dataset. This lack of information regarding these variables may have influenced the analysis.

### Conclusions, and implications of the study

Approximately three-quarters of the population in East Africa have access to improved drinking water, although the quality of water in the region is still considered poor. The source of drinking water in East Africa is associated with various factors such as the age and gender of the household head, wealth index, educational attainment, place of residence, types of toilet facilities, time taken to access the water source, community media exposure, and community poverty level. To address these challenges, it is crucial for state officials, non-governmental organizations, and local health administrators to collaborate and work towards improving the quality of drinking water. Special attention should be given to high-risk groups, including communities with high poverty and low literacy rates, poor households, and rural residents, in order to mitigate the negative consequences of using unimproved drinking water sources. Strengthening women’s empowerment is also recommended, and the use of mass media messages can be instrumental in promoting the adoption of improved drinking water sources in East Africa.

Despite the relatively high percentage of the population in East Africa having access to improved drinking water, the study highlights that the quality of water in the region is still rated as poor. Therefore, it is imperative for policymakers to prioritize initiatives that ensure the water from improved sources is not only accessible but also safe and meets quality standards. This can be achieved through investments in water treatment and monitoring systems.

The study identifies various socioeconomic factors that are associated with the source of drinking water. In order to address disparities and promote equitable access to safe water, policymakers should focus on implementing targeted interventions that specifically cater to vulnerable populations, including those with lower educational attainment, a lower wealth index, and those residing in impoverished communities. Furthermore, the findings suggest that factors such as the time required to access the drinking water source and the availability of proper toilet facilities are also associated with the source of drinking water. To address these issues, policymakers should prioritize infrastructure development aimed at reducing the time needed to obtain water and ensuring the availability of adequate sanitation facilities.

Researchers can build upon this study by conducting more comprehensive investigations to gain a deeper understanding of the reasons underlying the identified associations. For example, exploring the specific obstacles and challenges faced by different demographic groups in accessing improved drinking water sources can provide valuable insights for targeted interventions. Additionally, conducting longitudinal studies can aid researchers in evaluating the long-term impacts of interventions and policy changes. By tracking changes in access to improved drinking water and related factors over time, researchers can gather evidence to inform future policies and interventions.

Finally, policymakers, researchers, and stakeholders such as non-governmental organizations (NGOs) and community-based organizations can join forces to tackle the challenges at hand. By pooling together their resources and expertise, these stakeholders can develop and implement comprehensive strategies to enhance access to clean drinking water in East Africa. Community Engagement: It is crucial for stakeholders to actively involve communities in the decision-making process. By understanding the specific needs, concerns, and preferences of the communities regarding drinking water sources, stakeholders can design interventions that are tailored to the local context. This approach increases the likelihood of acceptance and sustainability of these interventions within communities.

## Data Availability

All data concerning this study are accommodated and presented in this document. The detailed data set can be freely accessible from the www.dhsprogram.comwebsite.

## References

[1] Hunter PR, MacDonald AM, Carter RC (2010). Water supply and health. PLoS Med.

[2] Gleick PH, Palaniappan M. Peak water limits to freshwater withdrawal and use. Proceedings of the National Academy of Sciences. 2010;107(25):11155-62. 10.1073/pnas.1004812107 PMC2895062 20498082

[3] Conant J. Sanitation and cleanliness for a healthy environment. Hesperian Foundation; 2005.

[4] Omole DO, Ndambuki JM (2014). Sustainable living in Africa: case of water, sanitation, air pollution and energy. Sustainability.

[5] Prüss-Ustün A, Bartram J, Clasen T, Colford JM, Cumming O, Curtis V (2014). Burden of disease from inadequate water, sanitation and hygiene in low‐and middle‐income settings: a retrospective analysis of data from 145 countries. Tropical Med Int Health.

[6] Supply WUJW, Programme SM. Progress on sanitation and drinking water: 2015 update and MDG assessment. World Health Organization; 2015.

[7] UNDESA U, UNECLAC U. Water for a sustainable world. World Water Development; 2015.

[8] Emenike P, Tenebe I, Omole D, Ndambuki J, Ogbiye A, Sojobi A, editors. Application of water recovery option for agricultural use in developing countries: Case study of a Nigerian community. Conference on International Research on Food Security, Natural Resource Management and Rural Development; 2015.

[9] Cassivi A, Johnston R, Waygood E, Dorea C (2018). Access to drinking water: time matters. J Water Health.

[10] Bain R, Cronk R, Wright J, Yang H, Slaymaker T, Bartram J (2014). Fecal contamination of drinking-water in low-and middle-income countries: a systematic review and meta-analysis. PLoS Med.

[11] Majuru B, Suhrcke M, Hunter PR (2016). How do households respond to unreliable water supplies? A systematic review. Int J Environ Res Public Health.

[12] Organization WH. Progress on household drinking water, sanitation and hygiene 2000–2020: five years into the SDGs. 2021.

[13] Forum WE. Wate Accessibility in sub Saharan Africar: https://www.weforum.org/agenda/2022/09/water-accessibility-divide-sub-saharan-africa-visualised/. 2022.

[14] Supply WUJW, Programme SM. Progress on drinking water and sanitation: 2014 update. World Health Organization; 2014.

[15] Tetteh JD, Templeton MR, Cavanaugh A, Bixby H, Owusu G, Yidana SM (2022). Spatial heterogeneity in drinking water sources in the Greater Accra Metropolitan Area (GAMA), Ghana. Popul Environ.

[16] Mills JE, Cumming O, UNICEF. The impact of water, sanitation and hygiene on key health and social outcomes. Sanitation and Hygiene Applied Research for Equity (SHARE) and. 2016;112.

[17] Saxena SK, Kumar S, Haikerwal A, Bhatt ML. Introductory chapter: neglected tropical waterborne infectious diseases-strategies for mitigation. Water Challenges Urbanizing World. 2018;1.

[18] Organization WH. Progress on drinking water, sanitation and hygiene: 2017 update and SDG baselines. 2017.

[19] Feleke H, Medhin G, Kloos H, Gangathulasi J, Asrat D (2018). Household-stored drinking water quality among households of under-five children with and without acute diarrhea in towns of Wegera District, in North Gondar, Northwest Ethiopia. Environ Monit Assess.

[20] Croft TN, Marshall AM, Allen CK, Arnold F, Assaf S, Balian S. Guide to DHS statistics. Rockville: ICF. 2018;645.

[21] Union A. Report on sustainable development goals for the Eastern Africa subregion.

[22] Croft T, Marshall AM, Allen CK, Arnold F, Assaf S, Balian S (2020). Guide to DHS statistics: DHS-7 (version 2).

[23] Goldstein H. Multilevel statistical models. Wiley; 2011.

[24] Liu X. Applied ordinal logistic regression using Stata: from single-level to multilevel modeling. Sage; 2015.

[25] Andualem Z, Dagne H, Azene ZN, Taddese AA, Dagnew B, Fisseha R (2021). Households access to improved drinking water sources and toilet facilities in Ethiopia: a multilevel analysis based on 2016 Ethiopian demographic and Health Survey. BMJ open.

[26] Simelane MS, Shongwe MC, Vermaak K, Zwane E (2020). Determinants of households’ access to improved drinking water sources: a secondary analysis of eswatini 2010 and 2014 multiple indicator cluster surveys. Adv Public Health.

[27] Mulenga JN, Bwalya BB, Chishimba KK. Determinants and inequalities in access to improved water sources and sanitation among the Zambian households. 2017.

[28] Agbadi P, Darkwah E, Kenney PL. A multilevel analysis of regressors of access to improved drinking water and sanitation facilities in Ghana. Journal of environmental and public health. 2019;2019. 10.1155/2019/3983869 PMC6589203 31275403

[29] Aragaw FM, Merid MW, Tebeje TM, Erkihun MG, Tesfaye AH (2023). Unimproved source of drinking water and its associated factors: a spatial and multilevel analysis of Ethiopian demographic and health survey. BMC Public Health.

[30] Huong LTT, Tuyet-Hanh TT, Minh HV, Ha BTT, Anh NQ, Huong NT (2020). Access to improved water sources and sanitation in minority ethnic people in Vietnam and some sociodemographic associations: a 2019 national survey. Environ Health Insights.

[31] Gomez M, Perdiguero J, Sanz A (2019). Socioeconomic factors affecting water access in rural areas of low and middle income countries. Water.

[32] Bogale GG (2020). Hotspots of unimproved sources of drinking water in Ethiopia: mapping and spatial analysis of Ethiopia demographic and health survey data 2016. BMC Public Health.

[33] Irianti S, Prasetyoputra P, Sasimartoyo TP (2016). Determinants of household drinking-water source in Indonesia: an analysis of the 2007 Indonesian family life survey. Cogent Med.

[34] Abubakar IR (2019). Factors influencing household access to drinking water in Nigeria. Utilities Policy.

[35] Mahama AM, Anaman KA, Osei-Akoto I (2014). Factors influencing householders’ access to improved water in low-income urban areas of Accra, Ghana. J Water Health.

[36] Adebola O (2019). Disparities in Access to Improved and Unimproved Sources of Drinking Water and Toilet Facilities in Nigeria: a Socio-economic dichotomy. J Sustainable Technol.

[37] Alexander CC, Shrestha S, Tounkara MD, Cooper S, Hunt L, Hoj TH (2019). Media access is associated with knowledge of optimal water, sanitation and hygiene practices in Tanzania. Int J Environ Res Public Health.

[38] Azanaw J, Abera E, Malede A, Endalew M (2023). A multilevel analysis of improved drinking water sources and sanitation facilities in Ethiopia: using 2019 Ethiopia mini demographic and health survey. Front Public Health.

[39] Tuyet-Hanh TT, Lee J-K, Oh J, Van Minh H, Ou Lee C, Hoan LT (2016). Household trends in access to improved water sources and sanitation facilities in Vietnam and associated factors: findings from the multiple Indicator cluster surveys, 2000–2011. Global Health Action.

[40] Armah FA, Ekumah B, Yawson DO, Odoi JO, Afitiri A-R, Nyieku FE. Access to improved water and sanitation in sub-saharan Africa in a quarter century. Heliyon. 2018;4(11). 10.1016/j.heliyon.2018.e00931 PMC6240801 30480156

[41] Akpakli DE, Manyeh AK, Akpakli JK, Kukula V, Gyapong M (2018). Determinants of access to improved sanitation facilities in rural districts of southern Ghana: evidence from Dodowa Health and demographic surveillance site. BMC Res Notes.

[42] Angoua ELE, Dongo K, Templeton MR, Zinsstag J, Bonfoh B (2018). Barriers to access improved water and sanitation in poor peri-urban settlements of Abidjan, Côte d’Ivoire. PLoS ONE.

[43] Abubakar IR (2017). Access to sanitation facilities among Nigerian households: determinants and sustainability implications. Sustainability.

[44] Usman M, Gerber N, Pangaribowo E. Determinants of household drinking water quality in rural Ethiopia. ZEF-discussion papers on development policy. 2016(220).

